# Supplementary material for: Alien woody plants are more versatile than native, but both share similar therapeutic redundancy in South Africa
Source: PLoS One. 2021 Nov 30;16(11):e0260390. doi: 10.1371/journal.pone.0260390 (PMC8631623; doi:10.1371/journal.pone.0260390)
Supplement: Attachment — Submitted filename: response to comments.doc [file pone.0260390.s006.doc]

Johannesburg 9^th^ September 2021

**Cover Letter**

**Dear Editor,**

First of all, we thank you for keeping the online resubmission open after such a long time despite your repeated requests to resubmit the paper. The Covid-19 pandemic has disorganized the normal way of academic life, and this explains that.

Second, we have now revised the ms following closely the comments of each of the 3 reviewers, and the response letter is provided below. We also provide a revised ms with track changes so that you can follow all our changes.

We thank you again,

The Authors

---

**Response letter to Reviewers' comments:**

**Reviewer #1:** The paper aims to test four important hypotheses of ethnobiology (non-random hypothesis, availability hypothesis, versatility hypothesis and diversification hypothesis) using data from woody medicinal plants from southeastern Africa. The publication of this study can be of great contribution to ethnobiology, mainly because it makes a broad analysis about the entry of exotic species in local pharmacopoeias. However, I suggest a major revision before the manuscript is considered for publication. I hope the authors consider trying to clarify some ideas. Below are my comments and suggestions for change.

*We thank the reviewer. We have now revised the ms. following closely the reviewer’s comments.*

**Reviewer # 1**
First, I felt a disconnection between the title and the ideas presented and discussed. The title makes us think that the work is about the redundancy of exotic species, but in fact it is about a greater scope of hypotheses that can explain such redundancy or the absence of it, among other things in the selection of medicinal plants. Redundancy and its implications, after all, is little discussed.

*Following the comments from all reviewers, we have rerun all the analyses excluding non-medicinal plants from the tests of versatility and redundancy hypotheses. The results changed slightly, and this informed the revision of the title as follows: “****Alien woody plants are more versatile than native, but both share similar therapeutic redundancy in South Africa”****.*

*We have also provided more information about redundancy hypothesis and its implications as suggested by the reviewer (see the second last paragraph of the discussion):*

Secondly, I think the hypotheses could be better presented in the background. The diversification hypothesis needs to be better defined, as it seems to be confused with the versatility hypothesis.

*These two hypotheses are linked but not the same: diversification hypothesis implies an increase in medicinal species richness due to integration of alien plants whereas versatility hypothesis implies multiple uses for a plant. This revision has been provided in paragraph 4 of the Background section.*

In addition, there is a lack of dialogue between the non-random hypothesis and the other hypotheses that deal with the entry of exotic species in local pharmacopoeias.

*The link between all hypotheses has now been clarified in the first paragraph of the Background section, and the revision is highlighted with track changes.*

Another point about this hypothesis is that, according to what I understand from the non-random hypothesis (and I may be mistaken, so the need to explain it better in the background), the number of medicinal plants in a given family is predicted to NOT increase linearly with the total number of species in the family, precisely because some families are over-utilized whilst others are under-utilized.

*It is actually the opposite: In its original form, the number of medicinal plants in a family is predicted to increase linearly with the size of the family. (see lines 74-77 of the revised ms with track changes).*

In the background the authors define the ways to measure availability, however, in the methodology, they apparently do not use any of the metrics presented to test this hypothesis. Instead, they redefine availability as the total number of countries where the plant occurs. I believe that a plant can occur in many countries but not necessarily be of high availability in those locations. I hope that the authors clarify this measurement, otherwise it would be necessary to rethink whether the study can really propose to test such a hypothesis.

*We have removed ‘availability hypothesis’ from the study.*

Regarding the test of the diversification hypothesis, the measure for redundancy is very interesting, but I was thinking that a low redundancy score can still reveal an overlap between native and exotic (for example, a disease treated only by two plants, presenting low redundancy, however including a native and an exotic plant). I suggest, in addition to this analysis, to compare the number of medicinal categories in which there are only exotic with those that exist both or only native, which would show that the exotic entered the medical system to fill the gaps (diversification hypothesis).

*We fully agree with the reviewer. We follow his/her suggestion and calculate the number of species (native and alien) used in each disease. The summary of the finding is in the Figure 1 below: i) there is no disease treated by only alien plants, ii) there is only one disease treated by only native plants, that is cancer, and iii) there are several diseases treated by both native and alien, e.g., respiratory disease, parasitic/infection control, etc. This confirms that alien woody plants are indeed not unique in local pharmacopoeias.*

**Figure 1.** Number of species used in the treatment of different diseases. Disease categories are as follows: 1. Headache_Nervous_Mental_Disease, 2. Oral_Dental_Diseases, 3. Body_Pain_Killer_Anti_Inflammatory, 4. Kidney_Diseases, 5. Fever_Malaria; 6. Diabetes; 7. Burns_Wounds_Injury_Scars; 8. Respiratory_Diseases; 9. Skin_Diseases; 10. Eye disease; 11. Ear disease; 12. Parasitic_Infection_Control; 13. Gastrointestinal diseases; 14. Obstetrics_Gynaecology_Diseases; 15. cardiovascular diseases; 16. Growth disorders; 17. Blood disorder; 18. Anti_Venom_Poison; 19. HIV/AIDS; 20. Cancer

On the third test for diversification, exposed in lines 255-258, I believe it can show how the exotic are more important in the medical system than native or vice versa, but it is not a measure of diversification. One of the consequences of the diversification hypothesis is that exotic species can first enter the medical systems to fill blanks (diversification hypothesis) and then their application can be extended to the other diseases already treated by the native plants, and this would result in a greater number of medicinal categories. In other words, this test is extremely important for the discussion about the unfolding of the diversification hypothesis, and it should present a good discussion, but it is not in itself a measure of diversification.

*We agree with the reviewer that comparing the number of diseases treated by alien versus native species is actually not a test of diversification hypothesis. We have rectified this in the revised ms: As opposed to our initial indication that we used three approaches to test the diversification hypothesis, we have now indicated that we used two (see first sentence in the section* ***Testing diversification and versatility hypotheses****). Also, in the third paragraph of the same section, we have now presented the comparison of the number of diseases treated by alien versus native species as an additional analysis to the diversification hypothesis and not as the diversification test.*

In general, the paper brings very interesting results, which perhaps deserve to be discussed further, presenting some possible hypotheses for future studies (such as the surprising highlight of the Poaceae family).

*Alternative scenarios are discussed in paragraph 3 of the discussion section in the revised ms.*

I believe that many ideas reached the conclusion without being previously discussed, such as the idea that exotic species did not enter to diversify, but to increase redundancy, which would contribute to resilience (I suggest reading Nascimento A.L.B. et al. (2015)* if the authors believe redundancy is an interesting idea to explain their results).

*We thank the reviewer for this suggestion. We have now discussed our main message on redundancy in the light of Nascimento et al. (2015): “Therapeutic redundancy hypothesis suggests that different species may provide similar medicinal uses to local people (Albuquerque and Oliveira 2007; Nascimento et al. 2015). Redundancy has some benefits to therapeutically redundant species, which is that, in theory, they suffer less use-impacts due to the diffusion of use pressure among a wider array of plants (Albuquerque and Oliveira 2007). However, a recent evidence shows that less therapeutically redundant species may not suffer from a greater use pressure (Coe and Gaoue 2021). From ethnobotanic perspective, the implication of redundancy is that the loss of some redundant species would not severely compromise local therapeutic practices (Nascimento et al. 2015). However, redundancy may not necessarily result in less use-impacts for versatile species or when a redundant species is of cultural preference by local people (Ferreira et al. 2012; Nascimento et al. 2015). In the present study, although alien plants show higher redundancy values than native plants, the difference is not statistically significant. This means that the proportion of alien plants that treat the same disease is similar to that of native plants. Alien plants are not unique in the study area; they are used to treat the same disease native plants treat except for cancer for which no alien plant is recorded. Further studies are necessary to tell the scenario (high or light use-pressure) that applies to the alien woody species included in the present study. Such studies may also include alien herbaceous species which may not necessarily show the same redundancy as the woody species since the latter may not primarily be introduced in the first place for medicinal purpose.*

Finally, I think it is important to add a section presenting the limitations of the study. In addition to the observations noted above, any ethnobotanical study based on other studies, whose data collection was carried out by different researchers, probably using different methodologies and rapport efforts, has limitations, which does not invalidate the results (lines 166-167: "a database of all ethnobotanical studies that ever took 167 place in Africa, country by country, since 1847 "). In addition, I think it is important to emphasize the importance of herbaceous species for traditional pharmacopoeias, and that if they were included in the analyzes, perhaps the results would be different.

*Data analyzed in the present study are not collected through experiments but through interviews, field surveys and group discussions over several decades. As such we do not expect any issues or limitations due to differences in methodologies used by researchers who collected the data. We would have expected such limitations if data were generated through field experiment; this is not the case here.*

*However, we agree with the reviewer that including herbaceous species may change the pattern reported in the present study; this is a limitation to the present study, and this has now been reported in the revised ms. This is highlighted in the Conclusion section as follows: “Future studies are also expected to test the therapeutic redundancy of alien herbaceous species which are not taken into consideration in the present study. We predicted a different outcome from that reported in the present study on woody plants since the latter may not primarily be introduced into South Africa for medicinal purpose unlike the herbaceous plants”.*

*Utilitarian Redundancy: Conceptualization and Potential Applications in Ethnobiological Research. In: Albuquerque U., De Medeiros P., Casas A. (eds) Evolutionary Ethnobiology. Springer, Cham. (2015) <https://doi.org/10.1007/978-3-319-19917-7_9>

**Reviewer #2**: The study intitled “Therapeutic redundancy of alien medicinal woody plants in the southern Africa’s region pharmacopoeia” contributes to the evaluation of hypotheses that seek to explain the selection of exotic species in different pharmacopeias on a regional scale. However, different aspects throughout the text need to be made clearer. Below, I highlight these points.

-In the introduction, the first sentence indicates “factors guide the selection of medicinal plants". Right after, the paragraph indicates “patterns of plant use” and “drivers shape local people plant knowledge and use". In this sense, I suggest to indicate more clearly that the hypotheses in the paragraph are related to the selection of medicinal plants.

*We agree with the reviewer and have indicated, in the Introduction, the link between all hypotheses tested and plant selection and integration into local pharmacopoeias. This revision reads as follows: “In the present study, we tested three interlinked hypotheses to explain the integration of alien plants into local medicinal flora: non-random selection, diversification, and versatility hypotheses. The link between these hypotheses can be justified as follows: humans decide to select a given alien plant to be introduced into a new environment (nonrandom selection hypothesis); once the species is introduced, the answers to the following questions determine its susceptibility to integrate local medicinal flora: Is the alien plant abundant or accessible to people (availability hypothesis)? Does it increase local medicinal plant species richness (diversification hypothesis)? Does it have additional uses, apart from medicinal uses, that may justify its selection and introduction to the new environment (versatility hypothesis)?”*

In addition, at the end of this paragraph, the following passage is indicated: "However, existing studies examined these hypotheses individually and this limits our global understanding of how multiple drivers shape local people plant knowledge and use [2]." However, the reference “2” contributes to assess theses hypotheses jointly, so it is necessary to highlight what remains to be done, even considering the study “2”.

*The reference [2] is for the last part of the statement which reads as follows “….understanding of how multiple drivers shape local people plant knowledge and use”. We have now referenced the first part of the statement that indicates that most studies focus on the test of just one hypothesis: “However, existing studies examined these hypotheses individually [Yessoufou et al. 2015; Muleba et al. 2021; Coe and Gaoue 2021; Gaoue et al. 2021]…..”*

-At the beginning of the second paragraph, I suggest deleting the following passage to make the text more objective: "rather, the number of medicinal plants in a given family is predicted to increase linearly with the total number of species in the family".

*We maintain this passage because it is the essence of the nonrandom hypothesis.*

-From line 84, the following passage is indicated: “In parallel, alien plants can also be introduced into a new geographic region primarily for diverse services, including food, construction materials, ornamental, etc. and then be used for medicinal purpose when the need to fill some therapeutic gaps in local pharmacopoeias arises." Next, it is stated that such a scenario involves the versatility hypothesis. However, the last passage: “…and then be used for medicinal purpose when the need to fill some therapeutic gaps in local pharmacopoeias arises" is not necessarily related to the versatility hypothesis, but rather to the diversification hypothesis. The diversification hypothesis in fact indicates that exotic plants are incorporated to fill local therapeutic gaps (Albuquerque 2006, mentioned in the manuscript) and this is not clearly indicated in the work of Bennett and Prance (2000, also mentioned in the manuscript). Although both hypotheses are indicated in the paragraph, it would be interesting to adjust the excerpts in which the hypotheses are presented for that the reader is not confused.

*We thank the reviewer for this clarification. However, whether an alien species is integrated into local medicinal flora because of its versatility or for diversification purpose, the outcome is the same: to fill some therapeutic gaps in local pharmacopoeias. The difference between versatility and diversification is that a versatile alien plant is used for various purposes (medicinal and beyond) but, through the diversification, treatment options and choices of plants have increased (diversified). We have revised that paragraph to bring about more clarity (see paragraph 3 in the revised draft).*

-In line 105, I suggest removing the term “theories”, because so far in the introduction, hypotheses have been presented.

*Done.*

-In the topic “Medicinal status and various use categories of plants”, it would be interesting to clarify whether inclusion and exclusion criteria were used to select documents in the search for literature on the uses of species. In this case, did the documents obtained undergo a methodological quality assessment? Sometimes, many papers in a region may interview a few people and this may lead to the failure to register certain plants that would have medicinal use in a community or region, which would have implications for the results of this research.

*We agree that differences in methodologies may influence data collection and results. The reviewer indicated that “Sometimes, many papers in a region may interview a few people and this may lead to the failure to register certain plants that would have medicinal use in a community or region”. It is to avoid that some medicinal uses may be left out that we did an extensive literature search with a comprehensive list of key words (as indicated in the method section) to minimize potential gaps in data collection of plant uses. Obviously, traditional knowledge evolves with time, and as such, what is recorded for the present study may be improved as new knowledge is available.*

-In line 211, it would be interesting to specify whether any criteria were used to organize diseases into 20 different categories.

*Disease categories were broadly grouped based on the human body part that is concerned by the disease. For example, the category ‘oral/dental disease’ refers to disease that concerns the mouth, and ‘kidney disease’ has to do with kidney, etc. We have now specified this clarification in the revision.*

-In line 231, I suggest changing “dependent variables” to “predictor” or “independent variables”.

*Done.*

-In line 272, would (53 species) actually be (53 families)?

*It is 53 families. We have amended this.*

-In line 280, I suggest starting the paragraph by presenting the proportion of over-utilized families for exotic species, just as it was presented for native species.

*Done.*

-In line 313, it is stated that “there was a support for versatility hypothesis such that native plants tend to have more use categories than alien". According to the argument presented in the introduction to the versatility hypotheses, wouldn’t it be the opposite result (exotic species being indicated for a greater number of categories than native) that would support the hypothesis? I have this same question in the discussion topic, on lines 365-370. Thus, I believe that the text can be adjusted.

*We agree 100% with the reviewer. Actually, in the initial MS, we made a mistake by including non-medicinal plants in the test of versatility, and this does not make any sense. We have rerun the test after excluding non-medicinal plants. The new results have been reported as follows: “Finally, there was a support for versatility hypothesis such that alien plants tend to have more use categories than native (β = -0.15±0.06, p=0.008).*

-In the discussion, there is an indication that over-utilized families are rich in secondary compounds, except the Poaceae family, which is an alkaloid-poor family. It would also be interesting to present in the results the under-utilized families (both for native and exotic species) and indicate in the discussion whether these families tend to have low richness of secondary compounds.

*The list of both over- and under-utilized families for native and alien plants is presented in Supplemental Information as Table S4.*

In addition, there are differences in the 10 over-utilized families of native and exotic species. It would also be the case to present a discussion on these findings.

*This is done in the discussion as follows: "There are, however, differences in the top 10 over-utilized families of native and alien species; this points perhaps to the evidence that alien plants did contribute to the diversification of local medicinal flora".*

-In line 377, I suggest replacing “(native versus native)” to (native versus exotic).

*Done.*

-At the end of the discussion, I missed an explanation about the findings indicating non-significant differences between the redundancy values and the number of treatments between native and exotic species.

*We have elaborated further on this in the discussion: “Therapeutic redundancy hypothesis suggests that different species may provide similar medicinal uses to local people (Albuquerque and Oliveira 2007; Nascimento et al. 2015). Redundancy has some benefits to therapeutically redundant species, which is that, in theory, they suffer less use-impacts due to the diffusion of use pressure among a wider array of plants (Albuquerque and Oliveira 2007). However, a recent evidence shows that less therapeutically redundant species may not suffer from a greater use pressure (Coe and Gaoue 2021). From ethnobotanic perspective, the implication of redundancy is that the loss of some redundant species would not severely compromise local therapeutic practices (Nascimento et al. 2015). However, redundancy may not necessarily result in less use-impacts for versatile species or when a redundant species is of cultural preference by local people (Ferreira et al. 2012; Nascimento et al. 2015). In the present study, although alien plants show higher redundancy values than native plants, the difference is not statistically significant. This means that the proportion of alien plants that treat the same disease is similar to that of native plants. Alien plants are not unique in the study area; they are used to treat the same disease native plants treat except for cancer for which no alien plant is recorded. Further studies are necessary to tell the scenario (high or light use-pressure) that applies to the alien woody species included in the present study. Such studies may also include alien herbaceous species which may not necessarily show the same redundancy as the woody species since the latter may not primarily be introduced in the first place for medicinal purpose”.*

In this case, in the last paragraph of the discussion, the following excerpt is indicated: “Also, dealing with diverse cultures in southern Africa, the selection or use of woody plants in the region might therefore differ...". It would be interesting to develop a little more that explanation. In the topic of the conclusion, there are moments when possible explanations are presented, considering scenarios (such as the resilience scenario) and evidence presented in the literature. So, I suggest expanding these explanations in the discussion topic and summarizing the information present in the conclusion.

*We have revised the discussion along the line proposed by the reviewer (see the last 2 paragraph of the revised ms).*

**Reviewer #3**: In this paper, the authors propose to explore the drivers of medicinal uses of woody plants in the Southern African region (including seven countries). They do so by testing four hypotheses about plant selection and use (non-random, availability, versatility and diversification). The large datasets used for the study are built from literature reviews (for plant uses, including medicinal) and available botanical datasets.
The hypotheses that are being tested are quite clear, the methodology is well described. The analyses that are proposed are conducted correctly - despite some issues with data, see below for details. The main results obtained are i) to show that medicinal plant use in this area is non-random, thus confirming the non-random hypothesis; ii) to identify some possibly over-used families of plants, which open ways for further research, and iii) to show that the three other hypotheses didn’t explain in a significant way the variations observed.
It is an interesting paper, covering a large area; there has been a lot of work involved in literature review and collection of data into coherent datasets.

*We thank the reviewer for finding merit in our work.*

There is, however, a number of issues that need to be addressed by the authors to make their paper more convincing, regarding the analyses, the datasets, the wording of conceptual elements, and the limits of the study, particularly in relation with the chosen scale of analysis.

*We have revised the ms as detailed below.*

Major comments
1. The authors should provide more justification about the choice of the study area: why selecting these seven countries, and not limiting the study to one country, or to one bioclimatic region, which would eliminate some flaws related to the large-scale, while keeping a dataset large enough for statistical analyses?

*We agree with the reviewer that large scale data collection for ethnobotanic studies may be source of some flaws. For example, on a large scale, there may be some protected areas that prevent access of people to some plants, and therefore impeding the development of medicinal knowledge on such plants or limiting the number of medicinal uses recorded due to some plants being protected away from people. However, in the present study, we focused only on plants for which traditional medicinal knowledge have already been collected for several decades. are developed by people. Also, although collecting data at small scale is important in ethnobotany because bias is limited, it is equally critical to investigate patterns of ethnobotanic behavior at large scale. One scale must not exclude the other especially when data are readily available for a region that shares similar flora and ethnic groups across geographies, e.g., southern Africa. Onus is on ethnobotanists to develop methodological approaches that can improve methodological approaches. We have no doubt such improvement will become reality as time goes.*

2. Many dimensions likely to account for plant use are not taken into account in this study (let’s just mention the socio-cultural background). This should be mentioned in a dedicated paragraph, as well as other limits to the study that need to be spelled out clearly.

*Although we agree that several factors, e.g., socio-cultural background, may shape medicinal knowledge, these factors are central to the formulation of any of the hypotheses tested in the present study. That’s why we still did not dedicate a paragraph, as suggested by the reviewer, to such factors. What we need is which plants are used and for what, irrespective of the socio-economic background of the users. However, we acknowledge that other limitations are important, e.g., the fact that we included only woody plants. We have acknowledged this and call for future studies to test these hypotheses with herbaceous medicinal plants. This will allow a generalization (or not) of the findings reported here.*

3. In the same line, while the methods to constitute the datasets are well explained, and show that this task has been carried out with a lot of attention, it is important to also acknowledge the potential limits of these datasets. For example, the sources used to list medicinal uses are likely to reflect only a part of the actual uses, or to be based on individual accounts only (as is sometimes the case with early ethnobotanical studies), thus reflecting only a part of what is happening “on the ground”.

*Once again, we agree with the reviewer on this. Medicinal knowledge is dynamic and can change over time. What we documented here is what we currently know and, to make this as comprehensive as possible, we consulted several sources which represent the state of our current knowledge. For most of these sources, collection efforts cover several decades.*

About the non-random hypothesis
4. The way to explain and refer to the non-random hypothesis and associated results in the paper could be clearer. To my understanding, there are two main “components” in this hypothesis, as it is found in the literature.
The first component is that there can be a trend, to be observed in the data, that the number of species with medicinal uses increases in relation with family size. This relation can be linear, logarithmic, and else.

*We agree, but the initial formulation of the hypothesis by Moerman predicts a linear relationship between number of species and family size (He fitted a simple* ***linear*** *regression model).*

However, there is a key point here: if the relation is strictly linear, then it means there is no differences between families, the proportion of medicinal plants being the same across families. It shows that the odds of a species to be medicinal is constant across families, which points towards randomness rather than non-randomness.

*We totally disagree with this interpretation. In the case of strict linear relationship, the constant proportion of medicinal plants in a family implies non-randomness rather than randomness. If families randomly contain medicinal plants, then the proportion of medicinal plants wouldn’t be constant across families.*

The second component is then to show, with the help of residuals or any other method, the differences across families, some hosting a higher number of medicinal plants than expected, some a lower. The first component alone is not sufficient; as used by Moerman 1979, it is interesting insofar as it allows to look at the residuals, at the differences between families, and the residuals only will “prove” differences between families (and the removal of families and genera with a strong deviation then lead to a better correlation coefficient - idem). The same point is underlined by Moerman & Estabrook 2003: medicinal uses are related to families - regardless of the size of the families.

*Again, we disagree with the reviewer: The first component is indeed the core (thus sufficient) of the definition of the nonrandom hypothesis. A strong significant correlation between number of medicinal plants and family size (irrespective of the shape of the relationships) is what is required to define the nonrandom hypothesis. What the reviewer calls the second component, i.e., the use of the residuals to distinguish between over- and underutilised, is simply a consequence of the non-randomness. The calculation of the residuals of the models fitted (linear or nonlinear) follows to distinguish which families contain more or less medicinal plants than expected, given the model. There wouldn’t be a need to calculate the residuals (the second component) if there is no correlation between number of medicinal species and family size.*

*In addition, the work of Moerman & Estabrook (2003) the reviewer referred to is based on simulated data and not real data. All the studies we are aware of and which used real dataset (not simulated) reported evidence of non-randomness based on the relationships between medicinal plant and family size.*

So I recommend rephrasing or nuancing the sentences dealing with this hypothesis, as showing that size of families is a predictor of the number of medicinal plants it hosts is not directly a confirmation of the non-random hypothesis “in full” (as differences between families are not revealed this way): abstract (l. 36-38), l.65-69, 112-113, and in the discussion and conclusion (l.321-322: the results don’t show that all large families contain more medicinal plants than expected, only some of them).

*Based on our arguments above, we maintain the definition of the hypothesis as formulated in the initial submission. However, we did highlight that the relationships between medicinal species and family size may not necessarily be linear (paragraph 2 of the revised ms).*

5. Many families included in the sample only host one species (out of 127 families, 41 are represented by only 1 species), and many families display zero values for medicinal plants.
The binomial regression models may be affected by a high number of zero counts (cf Zuur et al. 2010): I suggest to provide details about this aspect of the dataset and provide justification about the validity of the results.
(Zuur et al. 2010. A protocol for data exploration to avoid common statistical problems. Methods in Ecology and Evolution (1):3-14.)

*We thank the reviewer for raising this statistical question and suggesting Zuur et al. 2010 where solutions can be found. First of all, we did not fit a binomial regression but a negative binomial. Second, traditionally, 0 values are dealt with by log(x+0.001)-transforming data. However, log-transformation and other form of transformations have been showed to perform poorly on count data (see O’Hara & Kotze 2010 In Method in Ecology and Evolution, 1, 118-122). In their study, O’Hara and Kotze (2010) clearly demonstrated that “the quasi-Poisson and negative binomial models consistently performed well, with little bias”*. *Even Zuur et al. (2010) that the reviewer recommended also agreed on the use of negative binomial model when they wrote “In regression-type techniques, outliers in the response variables are more complicated to deal with. Transforming the data is an option, but as the response variable is of primary interest, it is better to choose a statistical method that uses a probability distribution that allows greater variation for large mean values (e.g. gamma for continuous data; Poisson or negative binomial for count data) because doing this allows us to work with the original data".*

About the versatility hypothesis
6. While the versatility hypothesis is introduced in the paper in relation with medicinal plants, the analysis seems to have been conducted with all plant species, including the ones that don’t have any medicinal use - no details are provided (l.260-262, and results 313-315). It would be useful to i) precise if only plants with at least one medicinal use were taken into account, and ii) if not, repeat this analysis with a subset including only plants with a documented medicinal use (826 out of 1400 according to table S2), to keep the analysis in line with the hypothesis it is aiming to test. The results and discussion sections related to this analysis should be changed accordingly.

*We totally agree here with the reviewer. It does not make sense that plants with no medicinal use be included in the analysis of versatility and therapeutic redundancy. We have now rerun all the analyses on medicinal plants only. We found evidence for significant difference in versatility (P=0.008) between native and alien plants. There is however, no such significant in term of therapeutic redundancy (P=0.59)*

***Versatility:***

\#	Estimate Std. Error z value Pr(>|z|)

#(Intercept)	1.502658	0.060441 24.861 < 2e-16 ***

#tot_nber_diseases	0.006953	0.006027	1.154 0.24866

#originNative	-0.158186	0.060367 -2.620 0.00878 **

***Redundancy:***
 Estimate Std. Error t value Pr(>|t|)

#(Intercept)	0.22873	0.05138	4.452 9.69e-06 ***

\# tot_nber_use_categories 0.00657	0.00747	0.880	0.379

\# originNative	-0.02132	0.04021 -0.530	0.596

About the datasets
There are discrepancies in the datasets, that need to be corrected, as well as the analyses depending on these datasets:

7. Table S1: some species are recorded as non-medicinal (col.4) while having medicinal uses counted (col.6): Ekebergia_pterophylla_OM3263 in Meliaceae, several eucalyptus species in Myrtaceae. The residuals in table S4 are probably wrong for these families.

*Discrepancies in the data have been corrected.*

8. Conversely, many species are recorded as medicinal in S1, col.4, but without any precise medicinal use counted in col.6 (408 species in total). The redundancy score is null for all these species. Given that among these 408, 30 are alien and 378 native, it introduces an important bias in the results: redundancy scores are skewed towards zero among the native species. The glm models and W tests testing the diversification hypothesis should be repeated, excluding the species with missing values - another solution would be to complete the data, which I understand can be challenging if the literature is not precise enough. In all cases the authors should mention clearly if they worked with a subset or the whole dataset, why, and how it affects the overall results of their study. The results and discussion sections related to this analysis should be changed accordingly.

*We thank very much the reviewer for pointing these discrepancies out. We have looked closely to the data, removed non-medicinal plants from the dataset and re-analyzed the data. Details are provided in the revised ms.*

Statistics
9. It would be useful to the readers to have a summary table providing the basic descriptive statistics of the variables used in the analyses (e.g. number of medicinal plants per family, average number of medicinal uses per species,...), in a table inserted in the text or in supplementary material. This would allow the reader to get a quick grasp on the structure of the datasets and on variables’ value distribution.

*Done*

10. In the result section, some basic descriptive analyses could strenghten the key results, and show that exploratory analyses have been conducted. I would suggest to present these first, then move to inferential analyses such as the binomial models. For example, for the non-random hypothesis, the correlation between family size and number of medicinal species should be tested, and results provided and eventually discussed (there is a strong - almost perfect - linear correlation in the case of native flora), before moving to binomial model results.

*The descriptive analyses are now presented in the result section.*

11. In the same line,in the case of diversification, the results of the Wilcoxon tests should be presented first, before the model results (l.298-311).

*We do not have Wilcoxon test anymore.*

12. A similar test should be done in the case of the availability hypothesis (fig.3, l.289-296).

*The availability hypothesis is no longer part of the ms.*

Other comments
13. Regarding the phrasing of the epistemological relation between the hypotheses and the facts they are dedicated to help understand (i.e. plant uses and plant-related knowledge): some sentences should be nuanced to acknowledge the fact that the hypotheses that are being tested in the paper are very general and cannot account by themselves alone for the complexity of human-plant interactions (as can be described e.g. in an ethnographical work). I therefore recommend rephrasing the sentences l. 54-55 and 97-98.

*Done*

14. Over-utilization as revealed in the results (i.e. as an output of binomial regression models) doesn’t account for the quantity of plants that are being harvested in the environment. So the results point towards a possible over-exploitation, but do not prove it in any way (a species can have many uses but being harvested in small quantities). I recommend nuancing the way this is expressed - e.g. l. 347-351.

*We mention over-utilization and not over-exploitation or over-harvesting. The term ‘over-utilization’ in ethnobotany is used to indicate that some taxa are subject to more uses than expected under a given model.*

15. The authors mention several botanical surveys carried out between 2008 and 2014 (l.138-148). However we don’t know to what extent these surveys contributed to the dataset. How many species or presence of species/families were documented and added during these? Would the datasets have been uncomplete without these? Moreover, if these surveys provided an important part of the botanical datasets, how has data on uses of these species been collected, in relation with the places they were collected in? If some species were previously unknown in some of these areas, one can expect a lack of data on uses and knowledge about them.

*We recorded plant and plant uses from various available sources so that missing information in one source can be complemented by information in another source. For example, Schmidt et (2007) documented knowledge for more than 10 years in the region. The first author also contributed through fieldwork and for 4 years to the data collection (2008-2014). We accumulated these information without recording what is present in one source and missing in another. So, we wouldn’t be able to provide the reviewer with such information.*

16. The title doesn’t reflect appropriately the content of the article.

*We have revised the title.*

17. Following PlosOne data policy, a table providing the details of medicinal uses per species should be made available to the readers.

*What the reviewer is asking for is in Table S2.*

Minor comments:

18. The reference 46 doesn’t seem to be the right one to be cited l.159.

*The reviewer is right. We have amended this.*

19. The table S.2 counts 826 species with a medicinal use, the S.1 displays 825.

*corrected*

20. The families Monimiaceae and Stangeriaceae don’t appear in the table S4, while being included in S1.

corrected

21. It would be useful to add a column with the results of the combined native + alien negative binomial model (for non-random hyp.) to the table S4.

*We do not find this necessary because the results of the negative binomial model are already reported in the text (otherwise, this would be a repetition).*

22. L.349-351: the residuals for the Zamiaceae is quite low (0,077, the lowest positive residuals value in the analysis), I wouldn’t interpret it as showing over-utilization.

*We get the point of the reviewer but remain the residual close to 1 as positive. In any case, it cant be interpreted as negative i.e., under-utilized.*

23. The reference to fig.3, l.292 is not adapted: the figure doesn’t illustrate results of the binomial model.

*We have deleted Figure 3 from the statement.*
